# An indicator of cancer: downregulation of Monoamine Oxidase-A in multiple organs and species

**DOI:** 10.1186/1471-2164-9-134

**Published:** 2008-03-20

**Authors:** Leszek A Rybaczyk, Meredith J Bashaw, Dorothy R Pathak, Kun Huang

**Affiliations:** 1 Department of Biomedical Informatics, The Ohio State University, Columbus, OH, USA; 2 Department of Psychology, Franklin and Marshall College, Lancaster, PA, USA; 3 Departments of Epidemiology and Family Medicine, Michigan State University, East Lansing, MI, USA; 4 Comprehensive Cancer Center Biomedical Informatics Shared Resource, The Ohio State University, Columbus, OH, USA

## Abstract

**Background:**

Identifying consistent changes in cellular function that occur in multiple types of cancer could revolutionize the way cancer is treated. Previous work has produced promising results such as the identification of p53. Recently drugs that affect serotonin reuptake were shown to reduce the risk of colon cancer in man. Here, we analyze an ensemble of cancer datasets focusing on genes involved in the serotonergic pathway. Genechip datasets consisting of cancerous tissue from human, mouse, rat, or zebrafish were extracted from the GEO database. We first compared gene expression between cancerous tissues and normal tissues for each type of cancer and then identified changes that were common to a variety of cancer types.

**Results:**

Our analysis found that significant downregulation of MAO-A, the enzyme that metabolizes serotonin, occurred in multiple tissues from humans, rodents, and fish. MAO-A expression was decreased in 95.4% of human cancer patients and 94.2% of animal cancer cases compared to the non-cancerous controls.

**Conclusion:**

These are the first findings that identify a single reliable change in so many different cancers. Future studies should investigate links between MAO-A suppression and the development of cancer to determine the extent that MAO-A suppression contributes to increased cancer risk.

## Background

One of the key goals in cancer research is to identify biological changes that distinguish normal tissue from cancerous tissue. A common approach to identifying oncogenes has been to assess gene expression in each type of cancer and compare it to non-cancerous tissue of the same organ. Comparisons within a single cancer type (e.g., breast cancer) or class (e.g., leukemia) have yielded potential oncogenic mechanisms that have been successfully used to develop therapeutic strategies for individual cancer types. For instance, real-time polymerase chain reaction (qPCR) research has found increased expression of oncogenes like c-myc [1] and decreased expression of tumor suppressors like Rb [2]. Western blotting has been used to show overexpression of functional erb-B2 in breast cancers [3] and ovarian cancers [4]. Unfortunately, comparing the data obtained from studies of individual types of cancer has resulted in only limited success at detecting consistent changes among different types of cancers. One such success is the identification of a mutation in p53, a protein responsible for repairing cellular DNA, which occurs in approximately 50% of all cancers [5]. The discovery of similarities among various cancer tissues is the first step in identifying a common mechanism that contributes to the development of cancer. Once a change is identified, appropriate therapeutic targets can be developed to help physicians identify at-risk individuals and improve patient care. Indeed, novel therapeutic strategies have been developed as a result of the extensive study of p53 [6].

Although best known as a neurotransmitter, only 1% of the tryptophan (Trp) derivative, serotonin (5-HT), is found in the nervous system. The remaining serotonin is found in the periphery and is active in the immune, circulatory, reproductive, musculoskeletal, and gastrointestinal systems [7]. Depending on receptor distributions, serotonin activity can promote or reduce apoptosis [8]. Our previous work explored the activity of serotonin in an array of pathologies, particularly those in which epidemiological data suggests gender differences [7]. We proposed that estrogenic effects on serotonergic function and receptor distribution could explain gender differences in pathologic incidence, as well as some of the effects of estrogen on breast cancer. Rather than being specific to breast cancer, the role of serotonin and its precursor Trp in cellular physiology suggests that the metabolic pathway of tryptophan and as a result serotonin metabolism may be involved in the promotion or progression of cancers in general. There is some literature supporting this hypothesis [9-17], but further research is needed to understand the exact relationship between Trp or its metabolites and cancer. Recently a mechanism was proposed by which catabolism of Trp by indoleamine 2,3-dioxygenase can be linked to immune evasion in tumor cells [13,18]. Other studies suggest that decreased serum tryptophan levels are predictive of poorer prognosis and quality of life in cancer patients[14]. For serotonin specifically, several studies have shown that the selective serotonin reuptake inhibitors (SSRIs) which prevent the reuptake of serotonin thus increase extracellular serotonin levels, have anti-cancer activity in cancer cell lines[17], decrease incidence of cancer in both animals [19] and humans [9], and can be used as a treatment for lymphoma/leukemia [20].

Using genechip technology to study multiple types of cancer simultaneously we can identify whether multiple cancers have similar gene expression changes [21]. Here, we analyzed an ensemble of cancer genechip datasets focusing on genes involved in tryptophan metabolism, which include serotonergic genes among them. We first compare gene expression between cancerous tissues and normal tissues for each type of cancer and then identify changes that are common to a variety of cancer types.

## Results

**Figure 1:**
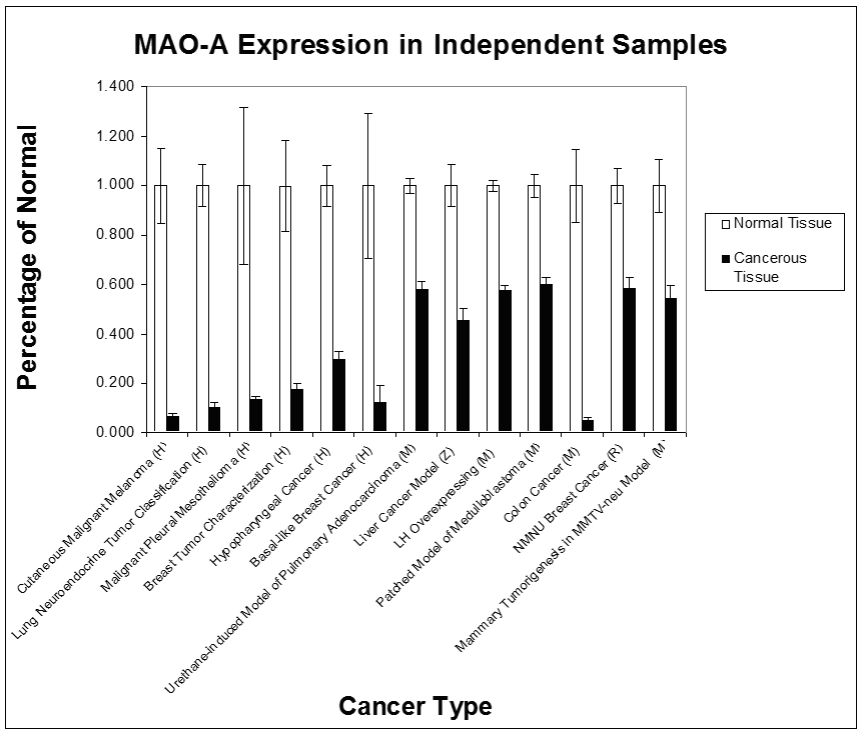
**Expression of MAO-A in normal and cancer tissue samples.** Tissues are from humans (H), mice (*Mus musculus*, M), rats (*Rattus norvegicus*, R), or zebrafish (*Danio rerio*, Z). Values are included for each dataset with independent samples in both human and animal models. Although all analyses were conducted on raw MAO-A expression levels, here we show both normal (white bars) and cancer (black bars) expression levels as a percentage of the mean expression in normal tissue within that data set. Control and cancer MAO-A expression levels are significantly different for all of the cancer types shown. Error bars indicate standard error of the mean.

By conducting a series of analyses focusing on tryptophan related gene expression data (see Table 1 for a list of genes analyzed) in the GEO database (gene expression omnibus) maintained by NCBI [22], we found that only Monoamine Oxidase A (MAO-A, E.C. 1.4.3.4) showed consistent decreased expression, in cancers among a variety of tissues from humans, rodents, and zebrafish. Specifically, only MAO-A expression was significantly altered in all 13 of the datasets that used non-cancerous patients as controls and half of the paired datasets. Table 2 provides specific p-values and the mean fold change in MAO-A for the datasets analyzed. Although the extent of downregulation varied among patients, cumulatively 95.4% of all of the tissue samples from human cancer patients, and 94.2% of all animal cancer cases showed lower MAO-A expression than the single lowest control sample in their respective dataset. Changes in expression for unpaired data are provided in Figure 1.

**Table 1:** Genes listed in the tryptophan pathway in KEGG.

**Gene symbol**	**Gene name**	**Gene symbol**	**Gene name**
AADAT	aminoadipate aminotransferase	HEMK1	HemK methyltransferase family member 1
AANAT	Arylalkylamine N-acetyltransferase	HSD17B10, HADH2	hydroxysteroid (17-beta-dehydrogenase)
ABP1	Amiloride binding protein 1	HSD17B4	hydroxysteroid
ACAT1, ACAT	acetyl-Coenzyme A acetyltransferase 1	INDO, IDO	indoleamine-pyrrole 2,3 dioxygenase
ACAT2	acetyl-Coenzyme A acetyltransferase 2	INDOL1	indoleamine-pyrrole 2,3 dioxygenase-like 1
ACMSD	Aminocarboxymuconate semialdehyde decarboxylase	INMT	indolethylamine N-methyltransferase
AFMID	Arylformamidase	KMO	Kynurenine 3-monooxygenase
ALDH1A3	Aldehyde dehydrogenase 1 family, member A	KYNU	kynureninase
ALDH1B1	Aldehyde dehydrogenase 1 family, member B	LCMT1	Leucine carboxyl methyltransferase 1
ALDH2	Aldehyde dehydrogenase 2 family	LCMT2	Leucine carboxyl methyltransferase 2
ALDH3A1, ALDH3	Aldehyde dehydrogenase 3 family	LNX1	ligand of numb-protein X 1
ALDH3A2	Aldehyde dehydrogenase 3 family, member A	MAOA	monoamine oxidase A
ALDH7A1	Aldehyde dehydrogenase 7 family, member A	MAOB	monoamine oxidase B
ALDH9A1	Aldehyde dehydrogenase 9 family, member A	METTL2B, METTL2	methyltransferase like 2B
AOC2	amine oxidase, copper containing 2	METTL6	methyltransferase like 6
AOC3	amine oxidase, copper containing 3	NFX1	nuclear transcription factor, X-box binding
AOX1	Aldehyde oxidase 1	OGDH	oxoglutarate
ASMT	acetylserotonin O-methyltransferase	OGDHL	oxoglutarate dehydrogenase-like
CARM1	Coactivator-associated arginine methyltransferase	PRMT2, HRMT1L1	Protein arginine methyltransferase
CAT	Catalase	PRMT3, HRMT1L3	Protein arginine methyltransferase
CYP1A1, CYP1	Cytochrome P450, family 1, subfamily A	PRMT5	Protein arginine methyltransferase 5
CYP1A2	Cytochrome P450, family 1, subfamily A	PRMT6, HRMT1L6	Protein arginine methyltransferase
CYP1B1, GLC3A	cytochrome P450, family 1, subfamily B	PRMT7	Protein arginine methyltransferase 7
DDC	dopa decarboxylase	PRMT8, HRMT1L3, HRMT1L4	Protein arginine methyltransferase
ECHS1	enoyl Coenzyme A hydratase, short chain, 1,	TDO2	Tryptophan 2,3-dioxygenase
EHHADH	enoyl-Coenzyme A	TPH1, TPRH, TPH	Tryptophan hydroxylase 1
GCDH	glutaryl-Coenzyme A dehydrogenase	TPH2	Tryptophan hydroxylase 2
HAAO	3-hydroxyanthranilate 3,4-dioxygenase	WARS, IFI53	tryptophanyl-tRNA synthetase
HADH, HADHSC	hydroxyacyl-Coenzyme A dehydrogenase	WARS2	tryptophanyl tRNA synthetase 2, mitochondrial
HADHA	hydroxyacyl-Coenzyme A dehydrogenase	WBSCR22	Williams Beuren syndrome chromosome region

Genes listed in the tryptophan pathway in KEGG. Each dataset was filtered for the tryptophan related genes and all tryptophan related genes included in the dataset were analyzed as described above. The column labeled Gene symbol contains the abbreviated gene symbol. The column labeled gene name contains the full gene name

**Table 2:** Descriptive information on datasets extracted from the GEO database used in this study.

**Human Independent Samples (Case-control)**							
GSE Number	GEO Description	Species	Control (N)	Cancer Type (N)	Mean Fold Difference in MAO-A	p-value	Percentage of cancer samples < lowest control

GSE3189	Cutaneous malignant melanoma	H	Normal (7)	Malignant Melanoma (45)	-15.0	4.9*10^-21^	100%
GSE1037	Lung neuroendocrine tumor classification	H	Normal Lung (19)	Small Cell lung Carcinoma (15)	-9.7	1.1*10^-10^	100%
GSE2549*	Human Malignant Pleural Mesothelioma	H	Normal Pleura (5)	Malignant pleural mesothelioma (40)	-7.4	4.6*10^-10^	90%
GSE61	Breast tumor characterization	H	Normal Breast (10)	Basal-like Tumors (10)	-5.6	1.6*10^-4^	100%
GSE2379	Hypopharyngeal cancer at various stages of progression	H	Normal Uvula (3)	Early Stage (4)	-3.4	7.7*10^-4^	100%
GSE3744	Basal-like breast cancer tumors	H	Normal Breast (7)	Basal-like Cancer (18)	-5.7	0.002	88%

* Lung tissue and cell lines were excluded. Only primary tumor samples and normal pleura were analyzed.							
**Paired Samples**							

GEO Series Number	Cancer Type	Species	Control (N)	Cancer (N)	Fold Difference in MAO-A	p-value	Percentage

GSE3268	Human Squamous Cell Carcinoma of the lung	H	Control (5)	Cancer (5)	-2.7	0.001	100%
GSE781	Clear Cell Carcinoma of the human kidney	H	Normal (7)	Renal clear Cell Carcinoma (7)	-2.2	0.002	100%
GSE2514	Pulmonary adenocarcinoma	H	Adjacent Normal (10)	Tumor (10)	-1.7	0.004	80%
GSE2685**	Gastric cancer	H	Normal (6)	Cancer (6)	-3.6	0.03^§^	100%
GSE3678	Papillary thyroid cancer	H	Normal (7)	Papillary thyroid Cancer (7)	-1.2	0.08^§^	71%
GSE3467	Papillary thyroid cancer	H	Normal (9)	Papillary thyroid Carcinoma (9)	-1.1	0.286^§^	67%

**Paired data was extracted and unpaired samples were excluded.							
**Animal Models**							

GEO Series Number	Cancer Type	Species	Control (N)	Cancer (N)	Fold Difference in MAO-A	p-value	Percentage

GSE2514	Urethane-induced lung tumor model of pulmonary adenocarcinoma	M	Adjacent Normal (10)	Tumor (29)	-1.7	6.6*10^-10^	86%
GSE3519***	Liver cancer model	Z	Normal Liver Tissue (10)	Liver Tumor (10)	-2.2	1.7*10^-5^	100%
GSE3348	LH overexpressing virgin mice (luteinizing hormone overexpression causes spontaneous mammary tumors)	M	Wild type Breast tissue (3)	LH-overexpressing Breast tissue (3)	-1.7	5.9*10^-5^	100%
GSE2426	Patched heterozygous model of medulloblastoma	M	Granule cell Precursor (4)	Tumor Cells from Heterozygotes (5)	-1.6	6.9*10^-5^	100%
GSE422	Colon cancer	M	C57/BL6 wild-type (6)	APC(Min/+) mutant (10)	-18.9	1.1*10^-4^	100%
GSE1872****	N-methyl-N-nitrosourea-induced breast cancer model (R)	R	Normal (11)	Cancer (9)	-1.7	5.9*10^-5^	100%
GSE2528	Mammary tumorigenesis in MMTV-neu model	M	Wild type Normal Breast (3)	Mammary tumors in MMTV-neu Model (7)	-1.8	0.001	100%

*** Zebrafish do not have separate MAO-A and MAO-B therefore MAO levels were analyzed.

**** Biological replicates were averaged for the analysis.

Descriptive information on datasets extracted from the GEO database used in this study. Cases are grouped by whether control and cancer tissues came from different human patients (independent samples), the same human patients (paired samples), or animal models. Tissues are from humans (H) mice (*Mus musculus*, M), rats (*Rattus norvegicus*, R), or zebrafish (*Danio rerio*, Z). For each dataset, sample sizes of control and cancer tissues are provided. The fold difference in mean intensities of MAO-A describes the amount of suppression of the expression of that gene in cancerous tissue; for example, a fold difference of -4 indicates 4 times less expression in cancer tissue than in normal tissue. P-values reflect significant fold differences in expression between cancer and control tissues using the appropriate t-test; cancer types are listed in order of decreasing significance of MAO-A expression. All datasets showed a significant change as determined by the sequential Bonferoni-Holm adjustment unless marked § The percentage column contains the percent of individual cancer tissue samples in each data set that had lower levels of MAO-A expression than the lowest single control sample.

Within each dataset, between 67% to 100% of patients had MAO-A expression below the lowest control sample (see Table 2 for individual values). Examining data from individual patients among the paired data revealed a remarkable pattern of downregulation in cancerous tissue among paired samples analyzed. Only a subset of the datasets that compared cancerous tissue to normal tissue from the same patient failed to show significant downregulation. The three datasets that did not contain a significant shift in MAO-A expression after correcting for multiple t-tests were the two papillary thyroid cancer datasets and one gastric cancer dataset. However 69% of thyroid cancer patients and 100% of gastric cancer patients exhibited a decrease in expression compared to non-cancerous tissue from the same patient, this discordance suggests that a less pronounced downregulation of MAO-A occurs in patients with these two types of cancer.

## Discussion

To our knowledge there has been no previous report of any genetic mutation or expression change that occurs with such high frequency among patients in a variety of cancers. The regularity of this change suggests that low levels of MAO-A might serve as a biomarker for cancer. In addition, it is possible that the lack of significant differences in thyroid and gastric cancer could be a result of an overall decrease in MAO-A levels in both normal and cancerous tissue. Unfortunately, datasets that compared cancerous thyroid and gastric tissues to those of healthy individuals were not available at the time of this study.

In higher species there are two isoforms of Monoamine Oxidase (MAO): MAO-A and MAO-B. In humans MAO-A preferentially metabolizes serotonin and the dopamine derivatives epinephrine, and norepinephrine; specifically MAO-A is responsible for catalyzing the inactivation of serotonin by removal of a terminal amine group. MAO-B metabolizes phenethylamine, and both MAO-A and MAO-B metabolize dopamine. There is a significant amount of literature regarding the role of dopamine in carcinogenesis [10,23,24], and several studies have indicated that it may have an antiproliferative effect [25]. The dopamine derivatives epinephrine and norepinephrine have also been implicated in carcinogenesis [26,27]. These hormones are released in response to stress and are also metabolized by MOA-A. Both epinephrine and norepinephrine are capable of increasing cell growth in tumor cells [28] and drugs that prevent norepinephrine signaling can reduce cancer risk [29]. In contrast the role of serotonin is less clear, while drugs that increase serotonin seem to have a protective effect in leukemias/lymphoms [20] as well as other cancers [12], increased serotonin appears to promote mitosis *in vitro *[11]. However our findings of a consistent change in MAO-A, but not MAO-B, in conjunction with the literature provide some support for our premise that the critical role of MAO-A in cancer occurs via the serotonergic system rather than the dopaminergic or catecholamine system.

MAO-A is the target of monoamine oxidase inhibitors (MAOIs), a class of antidepressants. There have been several epidemiologic studies that examine the risk between antidepressants and cancer[15,30-32]. While certain studies link the use of MAOIs with an increase risk for cancer[30,32] the literature remains inconclusive about the exact risk [15,30,32,33]. Given the downregulation of MAO-A observed in cancerous tissue here, one might expect that MAO-A-specific inhibitors may increase cancer risk. Indeed, MAO-AIs and other antidepressants increase cell proliferation in animal models [34-37]. If the same is true for humans, then MAO-AI's may be particularly likely to increase cancer risk.

SSRIs, another class of antidepressants, have recently been shown to be effective *in vitro *at inducing apoptosis in biopsy-like Burkitt's lymphoma cells, and have been shown *in vivo *to reduce the risk of colon cancer [9,38]. Visual inspection of data from precancerous tissue in MMTV-Neu and Patched heterozygote transgenic mice suggests that there is more severe downregulation in MAO-A expression in cancer than in the pre-cancerous condition. This study adds to the evidence that SSRIs may be a safer alternative than other antidepressants for treatment of depression in patients with a precancerous condition (such as Atypical Ductal Hyperplasia or Barrett's Esophagus) or cancer [16].

Since genechips measure mRNA levels within a complex sample we were unable to discriminate between actively dividing cells and those at rest. It is possible the downregulation of MAO-A observed occurs in the resting cells. Although the presence of downregulation in many cancers is compelling, this evidence is not adequate to establish causation, and further clinical studies are needed to determine whether MAOIs and specifically MAO-AIs cause an increase in cancer risk.

## Conclusion

Most current cancer research is focused on tissue-specific genetic mutations. Familial inheritance (e.g., APC in colon cancer), genetic mutation (e.g., p53), and overexpression of growth receptors (e.g., Her2-neu in breast cancer) can each lead to aberrant replication of a cell. Studies of these changes provide tremendous information about tissue-specific effects but are less informative about common changes that occur in multiple tissues. The similarity in the behavior of cancers from different organ systems and species indicates the potential for a universal change among cancers, regardless of the specific tissue or species. This study suggests that downregulation of MAO-A is such a change and could be an important indicator or even a factor in the development and spread of many types of cancers.

Interestingly, MAO-A inhibitors have been identified in tobacco [39]. If decreased expression of MAO-A is demonstrated in clinical trials to be a risk factor for development of cancer, it may be particularly important for individuals with low levels of MAO-A to be advised against smoking. Future studies should investigate links between smoking, MAO-A suppression, and the development of cancer to determine whether MAO-A suppression might be a mechanism by which smoking contributes to increased cancer risk. There are already previously published reports showing that PET scans can detect decreased MAO-A levels in smokers [40]. Our research shows that whole body PET scans might be a non-invasive way to identify MAO-A "cold spots", where localized downregulation indicates increased risk of cancer at that site. This type of a marker would permit more accurate diagnosis, leading to earlier treatment, and improved outcomes. Clinical studies are needed to determine whether changes in MAO-A can be used as a prognostic indicator of cancer risk in patients with a precancerous state.

Although the role of MAO-A in cancer has yet to be fully understood, examination of our results suggests its expression may act as a marker for the development of cancer. The presence of reduced expression of MAO-A in pre-cancerous states implies its levels indicate progression towards cancer, suggesting that MAO-A levels can be used to identify individuals that should receive increased surveillance and testing for the potential onset of cancer.

## Methods

19 Genechip datasets consisting of cancerous tissue from 10 different organs derived from human, mouse, rat or zebrafish were extracted from the GEO profiles database. Datasets were first identified by using the search terms "cancer" or "metastasis". All datasets in the GEO profiles database as of March 31^st ^2007 were considered. The genechip data was selected such that both control and cancer samples were contained in the same dataset. Because of the differences in gene expression that are inherent to cell culture and fixation [41,42], only data derived from non-processed primary biopsies was used. By definition cancer implies an invasive phenotype therefore all other tumor types were excluded such as adenomas, and carcinomas *in situ*. Among datasets that contained multiples types of cancer, the cancer with the closest number of samples to the control was used for analysis.

Table 2 describes the datasets used, which include 12 human cancer datasets, five mouse cancer datasets, one rat cancer dataset, and one dataset from zebrafish. All animal datasets were derived from cancers that were induced using viral, genetic, or by chemical means. No xenografts were included in this analysis. In total, 242 cancerous samples and 139 control samples were used. Prior to analysis, data that was logarithmic was transformed back to its original values, and all "null" values were excluded. Data points that were from more then one patient, also called pooled samples, were also excluded. Previous reports have shown that probe-sets can not be averaged [43] therefore all analyses were preformed on the probe-set with the highest mean expression value. Each genechip dataset was normalized (both intrachip and interchip) before being deposited in the GEO database. We independently validated the normalization in every dataset by inspecting the distribution of expression values. Datasets that were not appropriately normalized were excluded.

**Figure 2:**
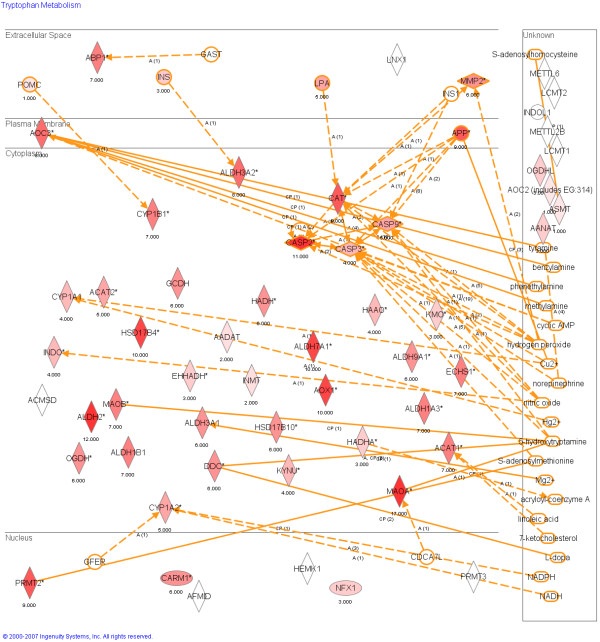
**A pathway representation of all the tryptophan related genes analyzed.** The figure was created by Ingenuity Pathway Studio, by importing the frequency of differential expression along with the gene name. The genes were then mapped and the frequency of differential expression was overlaid on top of the respective gene. The numbers below each gene represent the number of datasets in which a t-test of the gene's expression level resulted in p ≥ 0.05. The intensity of the coloration is provided to show respectively the number of datasets in which differential expression occurred. Gene names are capitalized and small molecules are lower case.

As our interest was in tryptophan-related genes, we examined all human, mouse, and zebrafish tryptophan pathway genes (~60 genes depending on the species and gene chip) listed in Kyoto Encyclopedia of genes and genomes (KEGG) using BRB-array tools [44] developed by the National Cancer Institute's Biometrics Research Branch (NCI BRB). Table 1 lists all the tryptophan genes in KEGG and Figure 2 is a graphical representation of the tryptophan genes included in this analysis, and the number of datasets in which these genes were differentially expressed. First, differences in the expression levels of the genes were examined within each of the individual cancer datasets. We performed either related samples or independent samples t-tests (as appropriate, see Table 2) on the change in expression of the genes between control and cancer samples for each dataset. Since we focused on the behavior of individual genes across multiple types of cancer rather than groups of genes in individual datasets we only compensated for the multiple t-tests over the composite number of datasets. The probability that we would observe the same false positive from among approximately 60 genes, in at least 16 out of the 19 separate datasets, is at most of the order of 7.8 × 10^-17^, an unlikely outcome. Therefore finding one specific gene (MAO-A) which is consistently down-regulated in 16 out of 19 datasets is an indication that the observed down-regulation of MAO-A in cancer tissues cannot be attributed to chance alone. Nonetheless, we did adjust for multiple t-tests among the 19 datasets by using the Bonferroni-Holm adjustment [45,46]. Specifically, we sorted the p-values for t-tests of expression of each gene from smallest to largest and compared the *i*-th p-value to the original alpha level (0.05) divided by the *number of data sets+1-i*. Thus we compared the smallest (first) p-value with 0.05/18 = 0.0028 and the largest (last) p-value with 0.05/1 = 0.05. For each dataset a list of tryptophan related genes whose expression level was significantly changed using these criteria was generated. Then the frequency of each gene appearing in all the lists was counted and the genes were sorted by the frequency from high to low. Only MAO-A was deregulated in the majority of the datasets analyzed.

## Abbreviations

MOA-A, Monoamine Oxidase A; qPCR, real time Polymerase Chain Reaction; 5-HT, 5-hydroxytryptamine or Serotonin; MAO-B Monoamine Oxidase B; GEO, National Center for Biotechnology Information Gene Expression Omnibus; KEGG, Kyoto Encyclopedia of genes and genomes; MMTV, Mouse Mammary Tumor Virus; MAO, Monoamine Oxidase; MAOI, Monoamine Oxidase Inhibitor; PET, Positron Emission Tomography, Trp, Tryptophan

## Authors' contributions

LR developed the original hypothesis for this work, conducted the analysis, participated in the development of the methodology, and contributed to the writing of the final version. MB participated in the development of the methodology, the writing of the manuscript, and validation of the data. DP participated in the validation of the statistics, and the writing of the manuscript. KH participated in the analysis, development of the methodology, validation of the data, and the writing of the manuscript. All authors read and approved the final manuscript.
